# Development and experimental analysis of a small integrated edge navigation sensor based on principle of circular arc array

**DOI:** 10.3389/fpls.2022.892388

**Published:** 2022-08-04

**Authors:** Binbin Xie, Jizhan Liu, Houkang Jiang, Lianjiang Cai, Lu Liu, Yuanxiang Li

**Affiliations:** ^1^Key Laboratory of Modern Agricultural Equipment and Technology, Jiangsu University, Zhenjiang, China; ^2^Institute of Technology, Anhui Agricultural University, Hefei, China; ^3^Intelligent Equipment Company of Hunan Xiangyuan Golden Spike, Loudi, China

**Keywords:** facility agriculture, corridor environment, edge walking, navigation sensors, miniaturization, integration, modularization

## Abstract

Signal, accuracy, and real-time performance of satellite, radar, and machine vision is a subject of concern in various complex agricultural environments. Therefore, the demand for a robust navigation sensor for indoor and vertical agricultural environment remains crucial, and it is a significant subject. In view of this, the relative edge pose detection method based on the ideal target band principle of the lateral center arc array, in this research, a small integrated arc array navigation sensor module based on adaptive detection arc technology, is developed, which costs about $100, autonomous edge navigation position, and attitude detection is realized in facility agriculture environment with continuous structured corridor or roadside features. In this research, a coupling method of reducing the radius of distance sensor arrangement, adjusting the unequal center angle, and increasing the detection distance is used to realize the miniaturization of the arc array arrangement. A semicircular modular rocket was designed to slide and adjust the center angle of the distance sensor, and the longitudinal installation position of the modularized sensor was adjusted by translating the circular arc of the detection; the convenient moving arrangement under different vehicle width and detection arc characteristics is realized. An adaptive construction method of detecting a circular arc based on self-calibrating detection distance of a distance sensor is proposed, which effectively reduces the difficulty of arranging the lateral central circular arc array; the fast construction of lateral detection arc is realized. In addition, in order to improve the accuracy and stability of the pose detection, the Mahalanobis distance algorithm and the standard Kalman filter are used to optimize the estimation of the ranging information and the relative pose of the edge. The experimental results show that the small integrated arc array navigation sensor module can independently construct photoelectric detection arcs with different characteristics to detect the position and attitude of the relative edge. When the road surface is concave and convex, the small integrated arc array navigation sensor module can still maintain the stable position and attitude detection of the relative edge for more than 30 s. In addition, when the walking speed of the autonomous navigation platform is 0.15 m/s to 0.35 m/s, the detection errors of lateral deviation and heading deviation relative to the road edge are less than 40 mm and 4.5°, respectively. The small integrated arc array navigation sensor module is less affected by the change of operating speed, and still has good accuracy and stability. The results show that the modularized edge navigation sensor has the advantages of fast and convenient use, high accuracy, and low cost; it can be applied to autonomous edge navigation control in greenhouse and plant and animal factories.

## Introduction

### Motivation and background

Facility agriculture planting makes crops unaffected by geographical environment, climate, and seasonal changes, guarantees crop yield, saves planting costs, and improves economic income benefits. Therefore, facility agriculture has received extensive attention and high-tech applications in countries around the world (Ferentinos et al., 2017; Soiket et al., 2019). Under the background that the world’s facility agriculture continues to develop toward high and new technologies, such as informatization, intelligence, and automation, by 2021, the total area of global facility agriculture has exceeded 4.6 million hm^2^, facility agriculture has developed rapidly, and the total planting area has continued to grow rapidly in China; the scale of facility agriculture has exceeded 4 million hm^2^, accounting for 85% of the global total. The autonomous navigation operation platform in the agricultural facility environment is mainly responsible for a large number of mobile operations, such as frequent handling, spraying, transplanting, harvesting, etc. Due to the complex, changeable, and more interference agricultural environment of the facility, mainstream autonomous navigation methods, such as satellite, radar, and machine vision, have the problems of weak signals, poor accuracy, and lagged real-time, which are difficult to meet operational requirements. Therefore, combining the characteristics of agricultural scenes, researching intelligent navigation technology and special sensors suitable for the facility agricultural environment is the key to realizing efficient autonomous navigation operations, and it is also a necessary condition for the development of unmanned facility agriculture (Liang et al., 2018; Soiket et al., 2019).

Autonomous navigation is the basis for the implementation of unmanned precision agriculture, which can effectively reduce labor intensity and improve operation accuracy and efficiency. In recent years, the rapid development of satellite autonomous navigation technology has been widely used in the field of agricultural environment. For example, Han et al. (2020) developed an agricultural machinery automatic driving system with multi-sensor data fusion algorithm based on GNSS, RTK, and motion sensors, etc.; the root mean square error of the path following navigation test is less than 9 cm. Takai et al. (2014) designed an autonomous navigation system for agricultural machinery with adaptive control algorithm based on RTK-GPS and inertial sensors, and the lateral deviation control error was less than 5 cm. Li et al. (2019) used the Kalman filter algorithm to fuse information from sensors, such as GPS, gyroscope, and electronic compass, to obtain accurate navigation data, and carried out experimental tests on the Tieniu 654 tractor and Lovol TG1254 tractor produced in China. Yunpeng et al. (2019) developed an automatic navigation system for agricultural machinery based on real-time dynamic positioning technology, and differential GPS technology, which can achieve continuous high-precision positioning and navigation under the condition of GPS signal stability. With the rapid development of differential RTK technology, satellite positioning technology represented by GPS and BDS has been widely used in agriculture, road transportation, and ship transportation for precise navigation, but the indoor environment of facility agriculture will seriously block satellite signals. The signal is discontinuous, and the satellite navigation fails.

In order to realize autonomous navigation in unstructured agricultural environment, radar and machine vision autonomous navigation technology has become the main research direction of agricultural autonomous navigation technology. For example, Jia et al. (2015) realized the edge detection of a flat greenhouse road based on a two-dimensional lidar, which has a poor adaptability to the flat slope and uneven road on both sides of the road. Wang et al. (2012) used machine vision to extract the sensitive area of the heating tube in the greenhouse ridge-planted tomato environment, and realized the autonomous navigation through the center baseline fitting between the ridges. Gao and Ming (2014) used K-means algorithm and the morphological erosion method to cluster and segment the collected greenhouse environment images, eliminate redundant interference information, and reduce the impact of illumination on machine vision recognition navigation paths. In the orchard tree row environment, corn and other high-stalk crops are planted in rows; lidar is often used to detect crop stalks to complete the line center baseline fitting and to achieve autonomous path perception and navigation in unstructured agricultural environments (Hiremath et al., 2014; Malavazi et al., 2018). In order to solve the problem of incorrectly fitting the navigation path and identifying obstacles in the unstructured environment where the distribution of path features is seriously uneven, the image features of the operating environment are recognized by machine vision to improve the accuracy of the navigation path fitting in complex environments, and can achieve rapid identification and classification of static and dynamic obstacles to improve autonomous navigation safety in unstructured environments (Yang et al., 2018; Inoue et al., 2019; Xu et al., 2021). Although radar and machine vision autonomous navigation technology has carried out many studies and achieved certain results in complex unstructured greenhouse environment and semi-natural interference conditions, such methods need to establish complex algorithms and are rich in information in facility agricultural environment. It is easy to be disturbed during the operation, and has not yet reached the point of application in actual production.

**FIGURE 1:**
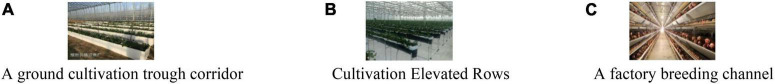
Modern standardized facility agricultural corridor environment.

**FIGURE 2:**
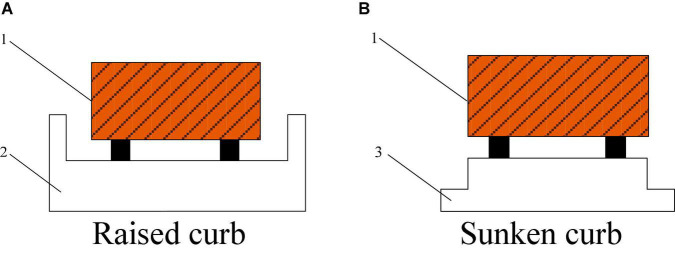
Characteristics of two types of curbs: (1) An autonomous moving platform, (2) A raised curb, and (3) A sinking curb.

**FIGURE 3:**
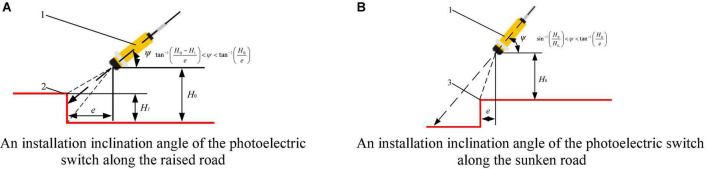
A layout principle of a photoelectric switch to identify the road edge: (1) A photoelectric switch, (2) A raised road edge, (3) A sunken road edge, *e*. A safety distance between the autonomous navigation platform and the curb line during driving (mm), *H*_0_. Height of the photoelectric switch installed on the mobile platform (mm), *H*_1_. Height of the raised curb (mm), *H*_*L*_. A range set by the photoelectric switch (mm), ψ. An inclination angle between a photoelectric switch and a road edge (°).

Recently, facility agriculture uses engineering technology and industrial production methods to provide a good growth environment for crops or animals. The environment of standardized greenhouse and plant-animal factory is usually equipped with furrows and cultivation troughs to form three-dimensional cultivation devices, presenting the characteristics of continuous structured corridors or roadsides, as shown in Figure 1. During the cultivation of fruits, vegetables, flowers and other plants, as well as the breeding of poultry and other animals, numbers of automatic operations, such as transporting, applying chemicals, transplanting and harvesting, should be accomplished by autonomous navigation platform in the corridor. So it is the current trend to use corridor or road-edge features to conduct curb line navigation technology research according to these features. The curbside characteristics of the environment of facility agriculture corridor, as shown in Figure 2. So, it is the current trend to use corridor or road edge features to conduct curb line navigation technology research according to these features. The curbside characteristics of the environment of facility agriculture corridor, as shown in Figure 2. The premise of autonomous edge navigation is to obtain the position information of the autonomous navigation platform along the relative road by using photoelectric switch. The photoelectric switch was installed on the side of the autonomous mobile platform walking along the edge, and judge the relative relationship between the autonomous mobile platform and the road edge based on the high-level and low-level changes of the photoelectric switch. In order to realize the detection of different road edge features, Ju (2017) adjusted the installation inclination of the photoelectric switch according to Figure 3, in combination with the geometric relationship between the convex edge and the sunken edge in Figure 2. During the operation of the autonomous mobile platform, when the horizontal distance to the curb is greater than or less than the safe distance, the high-level and low-level triggering states of the photoelectric switch will be changed to achieve real-time detection of the curb.

The realization of distance or collision perception based on mechanical contact, infrared photoelectric or ultrasonic sensors has been applied on many hair surfaces such as home and social service robots. However, they can only complete edge anti-collision or anti-drop control, and cannot accurately determine the relative position and posture of the autonomous navigation platform and edge, and cannot meet the requirements of high precision operation in facility agriculture (Zhao et al., 2012; Wang and Liang, 2015). In order to solve the above problems, many researchers have carried out the design of autonomous curb line navigation methods based on the road edge characteristics of the facility corridor environment. An ultrasonic or infrared photoelectric sensor is arranged laterally on the autonomous navigation platform to complete the distance measurement of the road edge to avoid collision, but the inclination state of the relative wall cannot be calculated and judged, causing the front or rear of the autonomous mobile platform to easily collide with the road edge, which is difficult to guarantee safe, efficient, and reliable autonomous driving along the road edge (Feng et al., 2012; Zhou, 2014).

For the problem that the single ultrasonic or infrared photoelectric sensor cannot obtain the heading deviation and transverse deviation accurately, multiple photoelectric switches are arranged in front of the autonomous mobile platform to measure the distance along the road, and multiple distance signals are used to construct fuzzy logic fusion to complete the curb line navigation. However, the calculation of this method is complicated, and the experimental results show that the walking posture is not stable enough (Xu et al., 2010; Yuan and Li, 2013). Du (2010) arranged several photoelectric switches in parallel lines on the side of the autonomous navigation platform to achieve autonomous walking along the road edge, but this method can only achieve a rough determination of the deviation state, and cannot complete the measurement of specific position and orientation, so it is difficult to ensure the smoothness of the curb line navigation and control accuracy.

**FIGURE 4:**
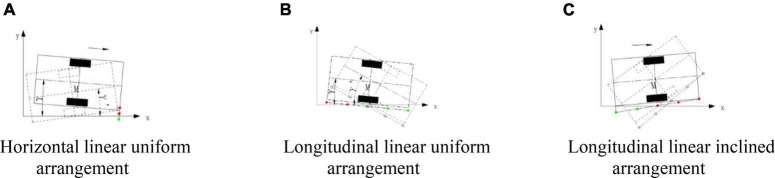
A linear arrangement scheme of a photoelectric switch array: γ. The heading angle of the autonomous mobile platform relative to the road along.

In order to improve the smoothness and control accuracy of edge navigation, Ju (2017) analyzed the arrangement of a photoelectric switch array, as shown in Figure 4. As shown in Figure 4A, due to the fixed lateral position of the photoelectric switch, the local horizontal discrete distance of the mobile platform relative to the road edge can be obtained from the photoelectric switch state. But, in the same photoelectric switch state, the heading angle of the mobile platform can change greatly, so the effective heading angle (γ) cannot be directly obtained from the current state of the photoelectric switch group. The information can only be obtained from the installation position of the photoelectric switch group, the time of state change before and after the photoelectric switch group, and the motion state of the mobile platform, and then derive the heading angle (γ) of the mobile platform through a certain algorithm, so it is difficult to provide more complete and effective information for the curb line navigation control of the autonomous mobile platform. As shown in Figure 4B, the horizontal discrete distance of the mobile platform relative to the road edge can be obtained, but it is difficult to obtain a more accurate heading angle, which cannot provide complete and effective information for the control of the autonomous mobile platform’s curb line navigation, As shown in Figure 4C, the horizontal discrete distance of the mobile platform relative to the road edge can also be obtained, but it is also difficult to obtain a more accurate heading angle, which cannot provide complete and effective information for the control of curb line navigation. To sum up, it is difficult to obtain the precise lateral deviation and heading deviation of an autonomous mobile platform relative to the road edge at the same time with the linear arrangement of photoelectric switches.

**FIGURE 5:**
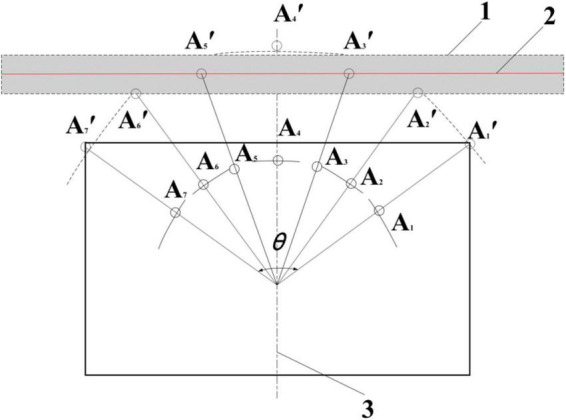
Seven principle of autonomous curb line navigation of a photoelectric switch arc array: (1) An ideal target belt, (2) A road edge, (3) An autonomous mobile platform, *A*_1_∼*A*_7_. A photoelectric switch, *A*_1_′∼*A*_7_′. A detection point of photoelectric switch, θ. The central angle of the photoelectric switch arc array.

In order to solve the problem that the precision lateral deviation and the course deviation from the autonomous mobile platform cannot be obtained with the linear arrangement of photoelectric switches, Ju et al. (2017) proposed an autonomous curb line navigation method based on seven photoelectric switches, took the trigger number of a photoelectric arc array signal and the number of the trigger center as dual indicators. The position and orientation detection model is established, and divides nine relative road edge position and orientation states according to the values of the dual indicators, and uses the fuzzy control method to realize real-time control of curb line navigation. As shown in Figure 5, under the detection arc of the photoelectric switch array set in the test, the control accuracy of the lateral deviation and the heading deviation between the autonomous mobile platform and the road edge were stable within the range of -35 mm∼15 mm and ±5°, respectively. The experimental results confirmed the feasibility of the method. However, this method needs to install and debug the photoelectric switch according to the actual vehicle body length and navigation accuracy requirements before use, which makes the use process cumbersome, and has not been deduced and verified for generalization and small integration, and has not yet entered the actual production operation. Therefore, building a small and integrated photoelectric switch arc array module can bring many conveniences into production practice, and can promote the development of low-cost and fast curb line navigation technology.

In response to this problem, this research developed a small integrated photoelectric arc array switch navigation sensor for facility corridor environment. The central photoelectric arc array based on the principle of the ideal target band, the system establishes a real-time position and an orientation detection method with unknown number of sensors. By adjusting the vertical installation position of the modular sensor through the shift detection arc method, the convenient mobile layout under different vehicle widths and detection arc characteristics can be achieved, which effectively reduces the difficulty of the lateral central arc array placement. Therefore, the small integrated circular arc array navigation sensor module can be widely used in many autonomous navigation platforms in the market, and can provide high-precision position detection. Specifically, the main features of the sensor are that it can realize convenient and fast curb line navigation with low cost and high precision. Specifically, the main features of the sensor are that it can realize convenient and fast curb line navigation with low cost and high precision.

### Scope and contribution

The overall goal of this research is to develop an intelligent navigation sensor suitable for the facility agricultural environment. Based on the ideal target principle of the lateral center arc array, we propose a relative edge pose detection method. We have developed a small integrated edge navigation module that adaptively constructs and detects arcs, and can realize low cost and fast edge navigation in the a facility agricultural corridor environment. The main contributions of this paper are as follows:

(1) In the early stage, it was verified the feasibility of a circular array edge-navigation method. In this paper, the edge pose state judgment rules and the pose deviation calculation method of an unknown sensor number were established, which can realize the autonomous edge pose detection under the configuration of different sensor numbers, arc center angles, and arc radiuses, and improve the universality of the arc array edge navigation method.
(2) In this article, a miniaturization scheme is proposed to reduce the sensor layout radius, adjust the non-equidistant center angle, and increase the detection distance. Under the condition of accurately reflecting the relative pose relationship between the head and the tail of the autonomous navigation platform and the road edge, and ensuring the resolution of edge pose detection and recognition, we realized the miniaturization of the arc array sensors.
(3) In order to realize the rapid adjustment and convenient arrangement of the navigation sensors along the edge, we propose an adaptive construction method of the detection arc based on the detection distance of the self-calibration sensor. According to geometric composition of the detection arc, a self-calibration equation of detection distance of sensors for each position and a calculation program of rapid arrangement of the arc center angle are established. The longitudinal installation position of the modularized sensor is adjusted by the method of the translation detection arc to match the installation position under different vehicle widths and detection arc characteristics.
(4) In order to improve the accuracy and stability of a relative edge position pose and enhance the performance of restraining the interference of environmental factors, we used the improved Mahalanobis distance algorithm to eliminate the abnormal ranging noise and environmental sudden change interference, and use the Kalman filter algorithm to optimally estimate the relative edge position and attitude state so as to achieve the purpose of weakening the relative edge position and attitude state detection error.
(5) Finally, we developed a low-cost small integrated arc array edge navigation sensor module by using embedded microcontroller and carried out experimental verification.

### Article structure

The rest of the research is organized as follows: the second part introduces the design scheme of the miniaturized optimized integrated arc array edge navigation sensor proposed in this study. The third part introduces the system design of a small optimized integrated arc array edge navigation sensor. The test design and results are presented in section “Experiment.” Finally, a summary of the study is provided in section “Results.”

## Materials and methods

In this section, we successively introduce the design architecture of the arc array edge navigation sensor module, the arc array edge navigation principle and universal modeling, the photoelectric arc array miniaturization integration scheme, the arc array adaptive self-calibration construction method, and the relative edge position and orientation detection error reduction method.

### Design architecture of a small integrated arc array edge navigation sensor module

**FIGURE 6:**
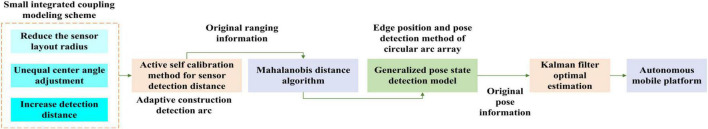
An overall architecture of a small integrated arc array edge navigation sensor module.

The essence of edge navigation is that the controller detects the relative pose deviation between the autonomous mobile platform and the continuous structured corridor or curb features in real time to correct the path. In order to solve the problems of a weak signal, poor accuracy and insufficient real-time performance of the current autonomous navigation technologies, such as satellite, radar, and machine vision in the facility agricultural environment, small integrated modules are used to achieve the purpose of accurate acquisition of the relative position and attitude along the edge of the autonomous mobile platform. In this paper, a general pose state detection model is built based on the relative edge pose detection method based on the ideal target band principle of the lateral central arc array in the author’s (Ju et al., 2017) previous research. A small integrated edge navigation sensor model is built by using the coupling method of reducing the sensor layout radius, adjusting the center angle of the non-bisection circle, increasing the detection distance. The active self-calibration sensor detection distance method is used to adaptively construct the edge pose detection arc, and the relative edge pose detection accuracy is improved by improving the Mahalanobis distance algorithm and Kalman filter algorithm. As shown in Figure 6, the development of a small integrated edge navigation sensor based on the principle of arc array is realized.

### The principle and universal modeling of arc array edge navigation

#### The principle of arc array edge navigation

**FIGURE 7:**
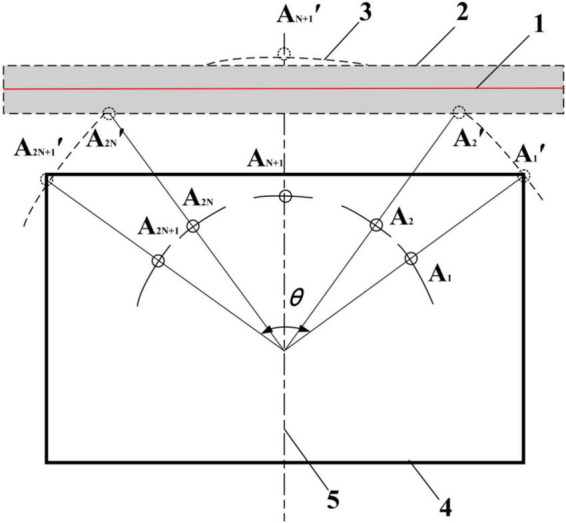
An autonomous edgewise navigation model of an N (odd) photoelectric switch lateral arc array: (1) A road edge, (2) An ideal target band, (3) A detection arc, (4) An autonomous mobile platform, (5) A transverse centerline.

The edge position and attitude detection of the arc array is to install an odd number of distance measuring sensors (2*N +* 1) on one side of the autonomous navigation platform in a centrosymmetric manner, and construct the edge position and attitude detection arc, as shown in Figure 7. The center position distance measuring sensor (*N* + 1, sensor detection point *A*_*N*+1_′) is aligned with the horizontal centerline of the autonomous navigation platform. In addition, the arc array edge navigation takes the width formed between the connecting line between the sensor detection point *A*_2_′*A*_2_*_*N*_*′ and the detection point *A*_2_*_*N*_*_–1_′*A*_2_*_*N*_*_+1_′ as the ideal target band for position and attitude adjustment, and controls the autonomous navigation platform to always move smoothly within the attitude range relatively stable with the edge of the road. At the same time, we can see that the position distribution of the detection points of each sensor determines the characteristics of different detection arcs.

#### Position and pose detection along the edge of the generalized arc array

**FIGURE 8:**
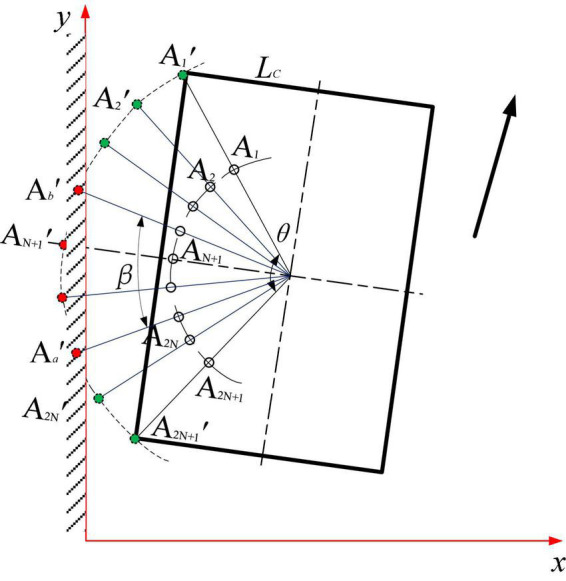
Calculation and analysis of pose deviation based on a photoelectric switch state.

In order to realize the universal edge pose detection of the arc array, establish the geometric relationship when the autonomous navigation platform has position and attitude deviation; as shown in Figure 8, the green dot represents the non-triggered ranging sensor, and the red dot represents the triggered ranging sensor. The edge pose state detection and judgment rules of unknown sensor number are established according to the previous research of the author (Ju et al., 2017), as shown in formula (1). The tolerance range of the calculation error of the heading deviation and transverse deviation of the autonomous navigation platform relative to the road edge can be further simplified, as shown in Formula (2). As shown in Table 1, the two parameters, signal trigger number *N*_*d*_ and trigger center serial number *N*_*f*_, are used to judge the relative pose state type of the autonomous navigation platform along the road. The larger the *N*_*d*_ is, the closer the autonomous navigation platform is to the road edge; on the contrary, the smaller the *N*_*d*_ is, the farther the autonomous navigation platform is from the road edge. *N*_*f*_ is the average value of the serial numbers of the photoelectric switches that are triggered when the road edge is detected outside the range of the photoelectric switch. The larger the *N*_*f*_ is, the more the head of the autonomous navigation platform deviates from the road edge; on the contrary, the smaller the *N*_*f*_, it means that the head of the autonomous navigation platform moves more toward the road edge.


(1) $$\left\{ \begin{array}{c} \tau =\frac{\theta }{2N}\\ N_{d}=b-a+1\\ N_{f}=\frac{b+a}{2}\\ \frac{\left(3+2N-b-a\right)\theta }{4N}<\gamma <\frac{\left(1+2N-b-a\right)\theta }{4N}\\ D=R\;\cos\frac{\beta }{2}-\left(R\;\cos\frac{\theta }{2}-L_{C}\right)\;\cos\gamma \\ \beta =\frac{\left(b-a+1\right)\theta }{2N} \end{array}\right.$$

**TABLE 1:** Generalized position and orientation state classification rules for unknown number of photoelectric switches.

Orientation	Position		
	1 ≤ *N*_*d*_<3	*N*_*d*_ = 3	3<*N*_*d*_ ≤ 2*N*-1
*N*<*N*_*f*_<*N*+1	A: Far away roadside to the outside yaw	B: Outside yaw	C: Near roadside to the outside
*N*_*f*_ = *N*+1	D: Far away roadside	E: Normal	F: Near roadside
*N*+1<*N*_*f*_<(4*N*+3)/2	G: Far away roadside to the inside yaw	H: Inside yaw	I: Near roadside to the inside yaw

In the formula: τ—The angle between adjacent sensor detection points; θ—The central angle of the circular arc; *N*—The total number of unilateral ranging sensors; *a*—Minimum serial number of the triggered ranging sensor; *b*—Maximum serial number of the range sensor; *N*_*d*_—Number of triggered ranging sensors; *N*_*f*_—Average value of serial number of triggered ranging sensor; γ—The heading deviation of the autonomous navigation platform relative to the road, °; *D*—Lateral deviation of the center point of the autonomous navigation platform relative to the road edge, mm; *R*—The detection radius of the photoelectric array switch, mm; *L*_*C*_—An autonomous navigation platform width half, mm.


(2) $$\left\{ \begin{array}{cc} \frac{\left(3+2N-2N_{f}\right)\theta }{4N}<\gamma <\frac{\left(1+2N-2N_{f}\right)\theta }{4N} & \\ R\;\cos\frac{N_{d}\theta }{4N}-\left(R\;\cos\frac{\theta }{2}-L_{C}\right)\;\cos\frac{\left(2+2N-N_{d}\right)}{4N}<D<R\;\cos\frac{N_{d}\theta }{4N} & \\ -\left(R\;\cos\frac{\theta }{2}-L_{C}\right)\;\cos\frac{\left(2N-N_{d}\right)}{4N} & \end{array}\right.$$

### A miniaturization scheme of the arc array edge navigation sensor

The relative edge position and orientation detection method based on the ideal target band principle of the lateral central arc array has been proved to be feasible by the author’s previous research. However, the arc array occupies a large proportion of space and the installation and layout of sensors are cumbersome. At present, the small-scale integrated system has not been developed, and the user-friendliness and practicality are insufficient. In this section, we carry out small-scale integrated scheme design for the purpose of convenient use.

#### Analysis and determination of a miniaturization scheme

**FIGURE 9:**
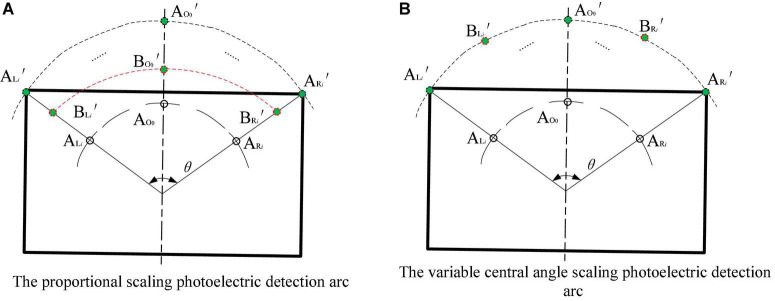
A schematic diagram of reducing the detection arc.

In essence, the miniaturization of the arc array is to reduce the plane layout size of the distance measuring sensor and realize the accurate acquisition of the position and attitude along the edge of the autonomous navigation platform. Through mathematical and geometric analysis, scaling the detection arc with an equal ratio column and scaling the detection arc with a variable center angle can reduce the layout size of the ranging sensor, as shown in Figure 9. The proportional column scaling arc in Figure 9A is to shrink A*_*Li*_*′ and A*_*Ri*_*′ inward to B*_*Li*_*′ and B*_*Ri*_*′ along the original radius direction without changing the center angle so as to reduce the arc radius and chord length of the proportional column so as to reduce the detection arc and achieve the purpose of miniaturization. When the longitudinal length of the autonomous mobile platform is large, the detection arc cannot reflect the relative position and orientation relationship between the head and the tail of the autonomous navigation platform and the road edge, resulting in a blind area along the curb line navigation, which cannot realize omni-directional detection and is not feasible. Figure 9B – the variable central angle scaling of the arc is to arrange the photoelectric switches A*_*Li*_*′ and A*_*Ri*_*′ on the original detection arc to the central position of the photoelectric switch under the condition that the radius of the detection arc remains unchanged. By changing the center-angle evenly distributed sensors, the layout chord length and the center angle of the photoelectric array are reduced so as to reduce the detection arc and achieve the purpose of miniaturization. The principle of this method is to move the detection point closer to the center of the detection arc, without changing the relative position and orientation relationship between the detection arc and the head and the tail of the autonomous navigation platform and the road edge, and there will be no blind area of curb line navigation.

**FIGURE 10:**
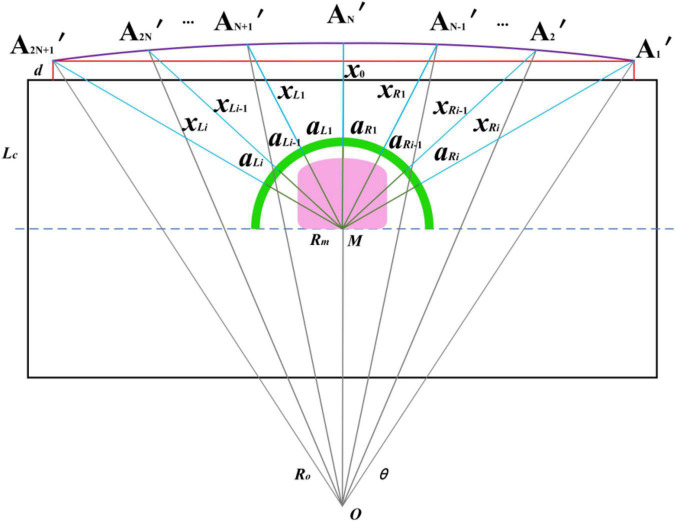
A coupling adjustment method of reducing the layout radius of ranging sensors, adjusting the center angle of non-equidistant circles and increasing the detection distance: *x*_0_. Distance detected by sensors at center positions, *x*_*Li*_. Distance detected by sensors at different positions on the left, *x*_*Ri*_. Distance detected by sensors at different positions on the right, *a*_*Li*_. The center angle of the sensor at different positions on the left, *a*_*Ri*_. The center angle of the sensor at different positions on the right, *R*_*m*_. A miniaturized arc array module original radius, *R*_*o*_. An original detection arc radius, A_1_′ ∼ A_2N+1_′. An ultrasonic sensor detection point.

According to Figure 8, it is found that the detection arc is formed by a series of curves connected by fixed points. In order to solve the problems in the sensor layout scheme of scaling, the detection arc with an equal ratio column and scaling the detection arc with a variable center angle, the two schemes are fused. The coupling adjustment layout method of reducing the ranging sensor layout radius - adjusting the unequal center angle - increasing the detection distance is adopted to realize the miniaturized layout design of the arc array, as shown in Figure 10. The semicircular shell with an arc chute (the green part in the figure) is designed. By adjusting the center angle and detection distance of different sensors, the original detection arc (the purple arc in the figure) is constructed by matching the detection points on the original detection arc to realize the miniaturization of the integrated arc array configuration. The pink part in Figure 10 is the embedded system integrated shell, and the green line is the semicircular shell radius of the arc chute, the blue line is the distance from the detection point of each position sensor, the horizontal red line is to detect the arc chord length, and the vertical red line is to detect the distance from the arc chord length to the side of the autonomous navigation platform.

#### Modular design of a circular arc array edge navigation sensor

**FIGURE 11:**
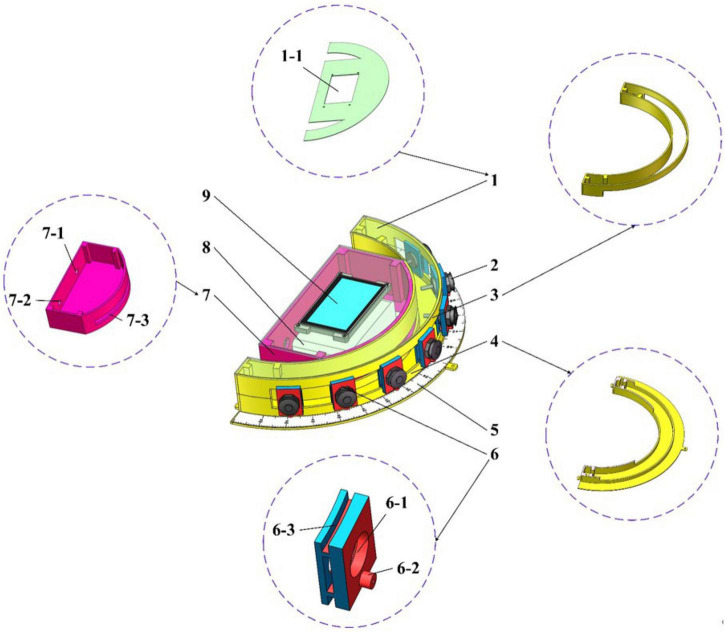
A structure of a circular arc array edge navigation sensor module: (1) An upper cover, (1-1) A man-machine interaction display mounting port, (2) A ranging sensor, (3) An arc chute half-round shell, upper part, (4) A lower part of an arc chute semicircular shell, (5) A calibration plate for central angle adjustment, (6) An angle-sliding adjustment plate, (6-1) A range sensor, (6-2) A bottom positioning bolt hole, (6-3) A chute, (7) Embedded System-integrated housing, (7-1) A power input port, (7-2) A navigation signal output port, (7-3) A range finder through a slot, (8) Embedded-controller housing, (9) A man-machine interaction display screen.

According to the arc array miniaturization integration scheme of reducing the distance measuring sensor layout radius – non-equidistant center angle adjustment – increasing the detection distance in Figure 10, the arc array edge navigation sensor module is designed. As shown in Figure 11, it consists of an arc chute semicircular shell, an embedded system integration shell, a center-angle adjustment calibration scale plate, an angle-sliding adjustment plate, an upper cover plate, a power input port, a navigation signal output port, and an installation positioning hole. In order to realize the matching of sensor detection points, fix the ranging sensor on the angle sliding adjusting plate to cooperate with the center angle adjusting calibration plate to slide freely to any angle alignment on the arc chute semicircular shell, lock the position of the sensor on the arc chute semicircular shell through the bottom positioning bolt, and adjust the sensor detection distance to complete the detection point setting. At the same time, the controller is installed in the embedded system integrated shell, and the arc surface of the shell is connected with the concave surface inside the semicircular shell of the arc chute to form a whole, which is conducive to the integrated sensor module design. In order to realize the human-computer interaction friendliness of the arc array edge navigation sensor module and facilitate the detection of arc parameters self-regulation, the upper cover plate with a serial port touch screen is designed, which can improve the power safety and water resistance. In addition, in order to reduce the interference of the power signal to the navigation signal transmission, the power line and the signal line are branched independently. The power input port and the navigation signal output port are placed at the tail of the embedded system-integrated shell and led out by the waterproof connector.

#### Modeling the detection distance and the central angle of sensors in different positions

**FIGURE 12:**
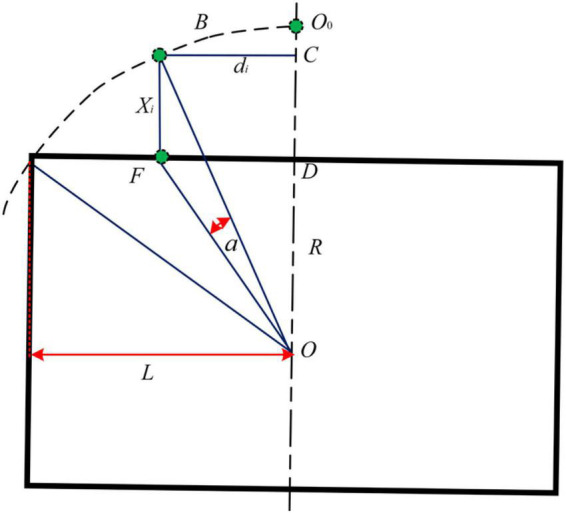
An analysis diagram of distance adjustment of a photoelectric switch detection point at each position.

It can be seen from Figure 11 that the arc array miniaturization integration scheme based on reducing the layout radius of the ranging sensors - adjusting the unequal center angle – increasing the detection distance is to match the detection points of the sensors by adjusting the center angle and detection distance of the sensors at different positions to complete the detection arc structure. Therefore, the center angle layout and detection distance adjustment modeling are carried out for the ranging sensors at different positions. Since there is an odd number of ranging sensors on the edge navigation sensor module of the circular arc array, and the *N* + 1 sensors about the central position are symmetrically distributed and equal in number. Therefore, only one side needs to be considered when modeling the center angle arrangement and detection distance adjustment of distance sensors at different positions. As shown in Figure 12, the center angle arrangement and the detection distance adjustment model are established with different position sensors on the left. Point *B* is the original position of the detection point of the *i*-th ranging sensor, point *F* is the position of the *i*-th ranging sensor on the side of the autonomous navigation platform, and *O*_*o*_ is the central position ranging sensor. The cosine value of ∠*BOF* of Δ*BOF* is solved by using the cosine formula of two-angle difference, and then the BF distance is solved by using the cosine theorem, as shown in Equation (3). Then, according to Figure 10, the detection distance of distance sensors at different positions is solved by using the Pythagorean theorem of the right triangle, and then the center angle of the layout of distance sensors at different positions is solved by using the cosine theorem, as shown in Equation (4).


(3) $$\left\{ \begin{array}{c} \theta_{i}=i•\frac{\theta }{2N}\\ d_{i}=R_{o}\left(1-\cos\frac{\theta }{2}\right)\;\tan\theta_{i}\\ \cos∠DOF=\frac{\bar{OD}}{\bar{OF}}=\frac{\sqrt{R_{o}^{2}-L^{2}}}{\sqrt{R_{o}^{2}-L^{2}+d_{i}^{2}}}\\ \sin∠DOF=\frac{\bar{DF}}{\bar{OF}}=\frac{d_{i}}{\sqrt{R_{o}^{2}-L^{2}+d_{i}^{2}}}\\ \cos∠BOC=\frac{\bar{BC}}{\bar{OB}}=\frac{\sqrt{R_{o}^{2}-d_{i}^{2}}}{R_{o}}\\ \sin∠BOC=\frac{\bar{OC}}{\bar{OB}}=\frac{d_{i}}{R_{o}}\\ \cos\left(∠DOF-∠BOC\right)=\cos∠DOF\cos∠BOC+\sin∠DOF\sin∠BOC\\ \cos\alpha =\cos\left(∠DOF-∠BOC\right)=\frac{\sqrt{R_{o}^{2}-L^{2}}}{\sqrt{R_{o}^{2}-L^{2}+d_{i}^{2}}}\times \frac{\sqrt{R_{o}^{2}-d_{i}^{2}}}{R_{o}}+\frac{d_{i}}{\sqrt{R_{o}^{2}-L^{2}+d_{i}^{2}}}\\ \times \frac{d_{i}}{R_{o}}\\ X_{i}=\bar{BF}\\ {\bar{BF}}^{2}={\bar{OF}}^{2}+{\bar{OB}}^{2}-2\times \bar{OF}\times \bar{OB}\times \cos\alpha \\ X_{i}^{2}=\left(R_{o}^{2}-L^{2}+d_{i}^{2}\right)+R_{o}^{2}-2\sqrt{R_{o}^{2}-L^{2}+d_{i}^{2}}R_{o}\cos\alpha \\ =2R_{o}^{2}-L^{2}+d_{i}^{2}-2\left(\sqrt{R_{o}^{2}-L^{2}}\times \sqrt{R_{o}^{2}-d^{2}}+d_{i}^{2}\right) \end{array}\right.$$

In the formula: θ—Detects the central angle of the arc, °; α—The reference calculation angle, °; *i*—Ranging serial number; *N*—Total number of unilateral ranging sensors; *θ_*i*_*—The original central angle of the unilateral i-th-ranging sensor, °; *d*_*i*_—The horizontal and vertical distance between the detection point of the i-th ranging sensor and the center position (the *N* + 1) ranging sensor, mm; *L*—The length of the autonomous navigation platform is half, mm; *R*_*o*_—An original detection radius, mm; *X*_*i*_—The vertical distance of the detection point of the *i*-th-ranging sensor, mm.


(4) $$\left\{ \begin{array}{c} X_{i}=\sqrt{2R_{o}^{2}-L^{2}-d_{i}^{2}-2\sqrt{\left(R_{o}^{2}-L^{2}\right)\left(R_{o}^{2}-d_{i}^{2}\right)}}\\ =\left(R_{o}^{2}-d_{i}^{2}\right)-\left(R_{o}^{2}-L^{2}\right)\\ L_{x}=2R_{o}\sin\frac{\theta }{2}\\ X_{0}=\frac{1}{\tan\left(90-\frac{\theta }{2}\right)}L_{x}\\ x_{i}=\left\{ \begin{array}{c} x_{i}=\sqrt{{\left(L_{C}+d+X_{i}\right)}^{2}+{\left(d_{i}\right)}^{2}}-R_{m}\;i0\\ x_{0}=L_{C}+d+X_{0}-R_{m}\;i=0 \end{array}\right.\\ a_{i}=90-\arctan\frac{L_{C}+d+X_{i}}{d_{i}} \end{array}\right.$$

In the formula: *R*_*m*_—The original radius of the miniaturized circular arc array module, mm; *d*—The vertical distance between the original detected arc chord length and the side of the autonomous navigation platform, mm; *L*_*C*_—Half width of the autonomous navigation platform, mm;*x*_0_. Distance detected by sensors at center positions, mm; *x*_*i*_. Detection distance of different position sensors.

#### Installation position under different vehicle width and test arc characteristics match

**FIGURE 13:**
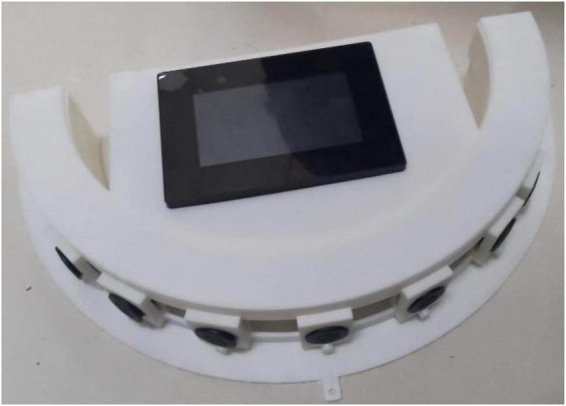
Physical drawing of a developed sensor.

According to the modular design scheme of the arc array edge navigation sensor shown in Figure 11, the lateral installation position of the edge navigation sensor module is the only one, and the longitudinal center line of the N + 1 sensor at the center position is always installed coincidently with the horizontal center line of the autonomous navigation platform. However, the relative position between the detection arc and the side of the autonomous navigation platform, the width of the autonomous navigation platform, and the distance between the detection arc chord length and the side of the autonomous navigation platform determine the longitudinal installation position of the edge navigation sensor module, but the bottom straight line of the edge navigation sensor module should always coincide with or be installed in parallel with the longitudinal centerline of the autonomous navigation platform. As shown in Figure 13, the arc array edge navigation sensor module is made with 600 mm as the original vehicle width and 30 mm as the distance from the original detection arc chord length to the side of the autonomous navigation platform. In Figure 13, the longitudinal centerline of the n + 1 sensor at the center of the arc array edge navigation sensor module coincides with the horizontal centerline of the autonomous navigation platform. To cope with the installation position matching of autonomous navigation platforms with different widths and detection arcs with different features, a method for adjusting the longitudinal installation position of the edge navigation sensor module is established, as shown in Equation (5). When *d_*L*_* < 0, the arc array moves along the edge navigation sensor module toward the direction of detecting the center of the arc; when *d_*L*_* > 0, the arc array moves along the edge navigation sensor module toward the detection arc direction.


(5) $$d_{L}=\left(w_{h}+d_{h}\right)-(L_{C}+d)\;\left\{ \begin{array}{c} d_{L}>0\text{Adjust}\text{in}\text{the}\mathrm{direction}\mathrm{of}\mathrm{arc}\\ d_{L}<0\text{Adjust}\text{towards}\text{the}\text{center}\text{of}\\ \text{the}\text{circle} \end{array}\right.$$

In the formula: *d*_*L*_—distance to be adjusted, mm; *w*_*h*_—actual width of the autonomous navigation platform, mm; *d*_*h*_—actual chord length vertical distance from the side of the autonomous navigation platform, mm; *d*—Original chord length vertical distance from the side of the autonomous navigation platform, mm; *L*_*C*_—Half the width of the original autonomous navigation platform, mm.

### Adaptive calibration and the construction method of detecting an arc

**FIGURE 14:**
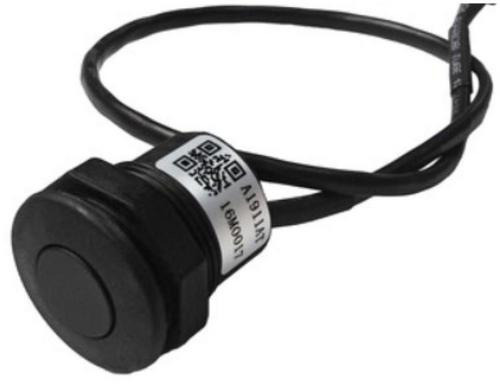
Physical drawing of a developed sensor.

**FIGURE 15:**
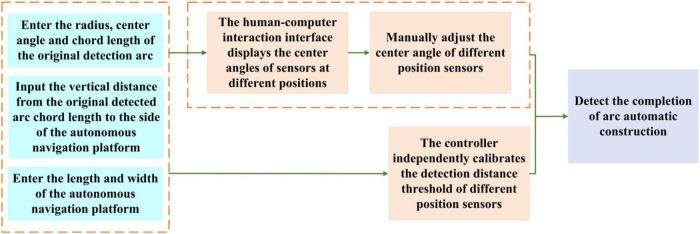
A schematic diagram of the construction method for detecting arc-adaptive calibration.

It can be seen from Figure 10 that the coupling adjustment layout method based on reducing the layout radius of the ranging sensor – adjusting the unequal center angle – increasing the detection distance is to realize the miniaturization design by adjusting the center angle and detection distance of the sensors at different positions. The angle of the circle center angle is quickly adjusted by sliding the angle-sliding adjusting plate equipped with the distance-measuring sensor at the position of the semicircular shell of the arc chute. Ju et al. (2017) used a switch type photoelectric switch to manually calibrate the detection distance, realizing the detection arc structure, resulting in cumbersome use, and the distance detection of the photoelectric switch is easily affected by environmental factors. In order to improve the convenience of using the arc array edge navigation sensor module, as shown in Figure 14, the PWM wave ultrasonic distance sensor (Dianyingpu DYP-A19-V1.0, Guangdong, China) is selected to detect the distance from the edge of the road. Based on the detection distance adjustment equation model of different position sensors, the trigger thresholds of different position sensors are automatically set to complete the automatic calibration of the detection points so as to realize the adaptive structure of the detection arc, as shown in Figure 15.

### Error analysis and reduction of edge-relative pose detection

#### Error analysis of edge-relative pose detection

**FIGURE 16:**
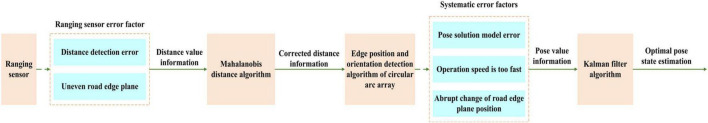
Multi-factor detection error compensation and a weakening scheme for an edge-relative pose.

The relative pose detection performance of the arc array edge navigation sensor module directly affects the operation accuracy of the autonomous navigation platform. In order to improve the edge operation accuracy of the autonomous navigation platform, the edge relative pose detection error must be reduced. In view of this, it is necessary to detect the source of the relative pose error of the arc array edge navigation sensor module, and take corresponding measures to reduce the error impact. According to the edge navigation principle of the circular arc array and the coupling adjustment arrangement method of reducing the layout radius of ranging sensors – adjusting the center angle of non-equidistant circles – increasing the detection distance, it can be seen that the relative pose detection errors are mainly caused by two reasons. On the one hand, it is caused by the distance detection error of the ranging sensor and the uneven road edge plane; on the other hand, it is caused by the position and attitude solution model error of the arc array navigation along the edge, the too fast operation speed and the sudden change of the road edge plane position. It can be seen that the relative pose detection error along the edge is affected by multi-factor coupling. In order to further improve the smoothness and stability of the arc array edge navigation sensor module, as shown in Figure 16, a multi-factor error comprehensive compensation scheme is adopted. First, the Mahalanobis distance algorithm is used to weaken the influence of the distance detection error of the ranging sensor and improve the accuracy and stability of the determination of the relative edge position and attitude. Then, the Kalman filter algorithm is used to estimate the relative position and attitude along the edge to improve the detection accuracy and anti-interference performance.

#### Reduction of sensor-ranging error based on Mahalanobis distance algorithm

**FIGURE 17:**
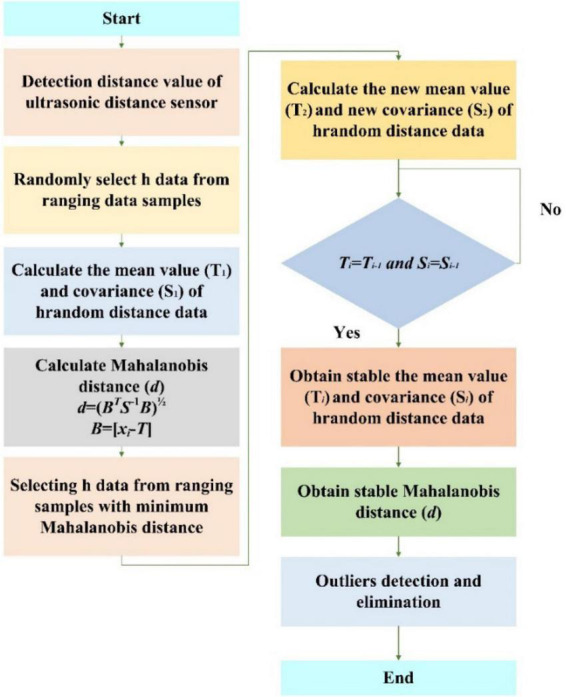
A flow chart of abnormal value Mahalanobis distance detection and the elimination method of the ultrasonic distance sensor: *d*, the Mahalanobis distance, *h*_*i*_, the sample size, *T*, the mean value of the sample data, and *S*, the covariance matrix of the sample data.

Because of the distance detection error and the uneven road edge plane, the ultrasonic distance sensor will have significant abnormal values in the distance measurement values, which will lead to the decline of the accuracy and stability of the determination of the relative position and attitude along the edge. In the process of ultrasonic sensor ranging, all ranging mean and covariance matrices are stable values calculated in the minimum covariance determinant estimation. Therefore, there is a significant difference between the Mahalanobis distance of abnormal values and the normal values in the calculated samples. Therefore, the Mahalanobis distance algorithm can be used to eliminate the interference of abnormal values of ultrasonic ranging sensors. In order to weaken the influence of environmental factors and the ultrasonic ranging sensor’s own factors on ranging error, the arc array edge navigation sensor module uses Markov distance algorithm to detect and eliminate the abnormal ranging value of the ultrasonic ranging sensor. However, in the standard Mahalanobis distance algorithm, Mahalanobis distance is removed by calculating the mean sum and covariance matrix of the original ranging data. When the sample size of the distance measurement data is small and there are many outliers in the ranging, the standard Mahalanobis distance algorithm will make the calculated mean value and the covariance matrix of the original ranging data deviates from the outliers, resulting in the outliers being detected as normal values, which will lead to the incomplete elimination of the outliers. In view of this, the fast minimum covariance determinant algorithm is adopted to obtain a stable covariance matrix and a stable average vector, and then the stable Mahalanobis distance is calculated according to the standard Mahalanobis distance equation to eliminate the outliers of distance detection, as shown in Figure 17, in which we set the threshold of Mahalanobis distance approximated to the chi-square distribution, ranging numerical sample data as a constant. When the Mahalanobis distance of the calculated ranging value is greater than the set threshold, it will be regarded as an abnormal value and will be discarded immediately.

#### Optimal estimation of a pose state based on the Kalman filter

**FIGURE 18:**
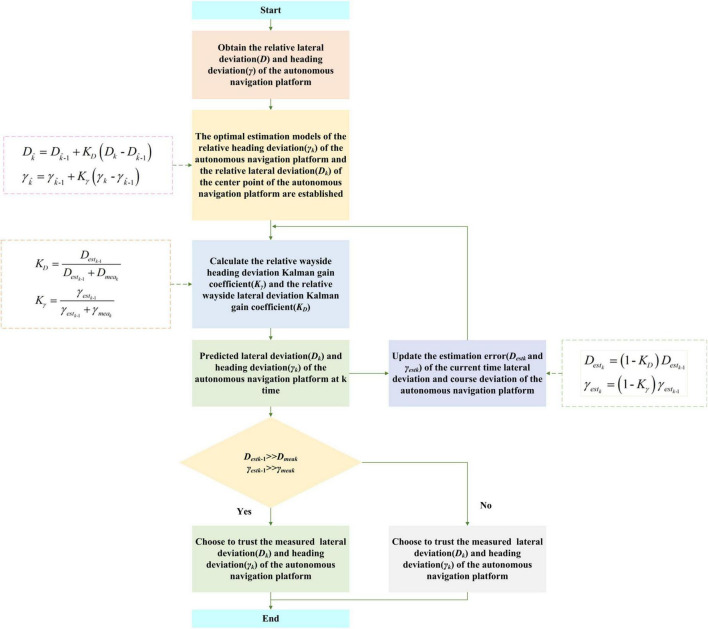
Kalman filter optimal estimation flow of a relative wayside pose: *D*_*k*_. The observed lateral deviation of the current autonomous navigation platform relative to the road edge. ${\text{D}}_{\hat{\text{k}}}$, the estimated lateral deviation of the current autonomous navigation platform relative to the road edge. ${\text{D}}_{\hat{\text{k}}-1}$, estimated value of the lateral deviation of the autonomous navigation platform relative to the road edge at the last moment. K_X_, Kalman gain coefficient of relative lateral deviation of the autonomous navigation platform. *γ_*k*_*, the observed heading deviation of the current autonomous navigation platform relative to the road. $\gamma_{\hat{\text{k}}}$, estimated heading deviation of the current autonomous navigation platform relative to the road edge. $\gamma_{\hat{\text{k}}-1}$, estimated value of the heading deviation of the autonomous navigation platform relative to the road edge at the last time. K, Kalman gain coefficient of relative heading deviation of the autonomous navigation platform. *D*_*estk*–1_, the estimation error of the lateral deviation of the autonomous navigation platform relative to the road edge at the last moment. *D*_*meak*_, the current autonomous navigation platform-relative lateral deviation measurement error along the road. *γ_*estk*_*_–1_, the estimation error of the heading deviation of the autonomous navigation platform relative to the road at the last moment. *γ_*meak*_*, the current autonomous navigation platform relative along the road heading deviation measurement error. *D*_*estk*_, estimation error of lateral deviation of the autonomous navigation platform relative to the road edge at the current time. *γ_*estk*_*, the estimated error of the relative heading deviation of the autonomous navigation platform at the current time.

The model error of pose solving for circular arc array edge navigation, too fast operating speed, and abrupt position along the road plane will further affect the position and attitude state detection accuracy. In order to ensure the operation accuracy of the autonomous navigation platform, the original position and attitude state detection information needs to be optimally estimated. Kalman filter algorithm is a recursive estimation algorithm in essence. It does not need to record observations and estimated historical data. It estimates the optimal value of the current state according to the estimated value of the last time state and the observed value of the current state in the system. It is widely used in the field of autonomous navigation and control. In view of this, the standard Kalman filter algorithm is used to smooth the relative position and attitude state information along the edge, improve the position and attitude detection accuracy and anti-interference performance, and weaken the influence of system error and environmental error, as shown in Figure 18. The specific steps are as follows: the first step is to detect the relative wayside pose data of the autonomous navigation platform; the second step is to establish the optimal estimation model of the relative heading deviation of the autonomous navigation platform and the relative lateral deviation of the center point of the autonomous navigation platform; Step 3: calculate the Kalman gain coefficient K_γ_ of the relative wayside heading deviation γ and the Kalman gain coefficient K*_*D*_* of the lateral deviation of the relative path edge; the fourth step is to measure and predict the relative wayside pose of the autonomous navigation platform at time k (the current time) according to the original autonomous navigation platform’s relative wayside pose at time k-1 (the previous time); the fifth step is to update the current time relative wayside lateral deviation estimation error and the current time relative wayside heading deviation estimation error for the next time relative wayside position and attitude information prediction of the autonomous navigation platform.

## Integrated embedded module design

The arc array edge navigation sensor is designed to realize the real-time detection of the relative position and attitude of the autonomous navigation platform and the output of navigation control information through modular packaging. Therefore, on the basis of the miniaturization scheme of reducing the radius of the ranging sensor, adjusting the non-equal center angle and increasing the detection distance, it is necessary to design the hardware circuit and software program of the sensor system. In this section, we introduce the sensor module hardware system design and the sensor software program design, in turn.

### Hardware design of a sensor module

**FIGURE 19:**
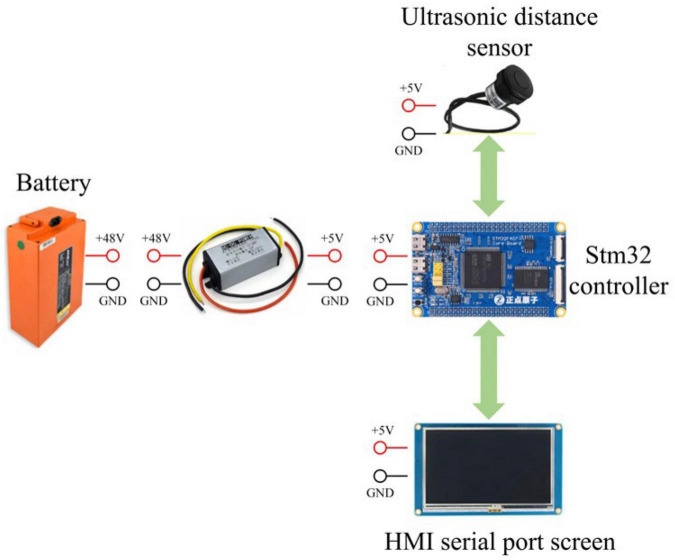
Hardware system composition of an arc array edge navigation sensor module.

In this paper, the arc array edge navigation sensor module is designed to accommodate up to 9 ultrasonic ranging sensors, as shown in Figure 19. In order to improve the anti-interference performance of environmental factors and realize the adaptive calibration structure of the detection arc, a PWM wave ultrasonic ranging sensor (Dianyingpu DYP-A19-V1.0, with the optimal accuracy of 1 mm, Guangdong, China) is selected to detect the distance from the road edge. The sensor uses the pulse width change time to measure the distance. It needs to use a microsecond timer to measure the pulse width time to complete the distance detection. At present, the commonly used PLC logic controller can only achieve millisecond timing. The number of timers of a 51 single-chip microcomputer controller is small and cannot support more than three ultrasonic distance sensors. However, there are more than eight timers in the STM32 MCU controller. In order to accommodate the distance measurement of nine ultrasonic ranging sensors at the same time, and increase the expansion performance of the arc array edge navigation sensor module. In this paper, stm32f407 embedded controller (punctual atomic core board, Guangdong, China) is selected to develop the hardware system of the sensor module. The controller built in 14 timers. In order to improve the user-friendliness of the arc array edge navigation sensor module and realize the adaptive structure of detecting arcs, an HMI serial port display screen (4.3-inch, four-wire system, Guangdong, China) is added to the sensor module hardware system. The center angle, arc radius, and photoelectric switch number of the original detected arcs can be input by touch, as well as the length and width of the autonomous navigation platform, and complete the automatic calculation of the adjustment parameters of the center angle arrangement of different position sensors and the independent calibration of the distance between detection points. The output of relative path edge position and attitude state of the autonomous navigation platform is the key to the design of the arc array edge navigation sensor module. The TTL serial port circuit is set on the hardware system of the sensor module, which can realize the position and attitude state and position and attitude deviation value in the form of RS232 or RS485 serial ports through different external modules so as to meet the control requirements of different lower computers. The cost of the arc array edge navigation sensor module designed in this paper is about US $100. As shown in Table 2, compared with the current laser radar and a visual camera, its price is relatively lower and its use is simpler.

**TABLE 2:** Manufacturing cost of an arc array edge navigation sensor module.

Type of sensors				
**Arc array edge navigation sensor module**			**Lidar**	**Visual camera**
**Part name**	**Unit cost/$**	**Price range/$**	**Price range/$**	**Price range/$**
Plastic shell	30	<100	>300	>400
STM32 controller	25			
HMI serial port screen	30			
Ultra sonic distance sensor	5			
Power conversion module	10			

### Software design of the sensor module

**FIGURE 20:**
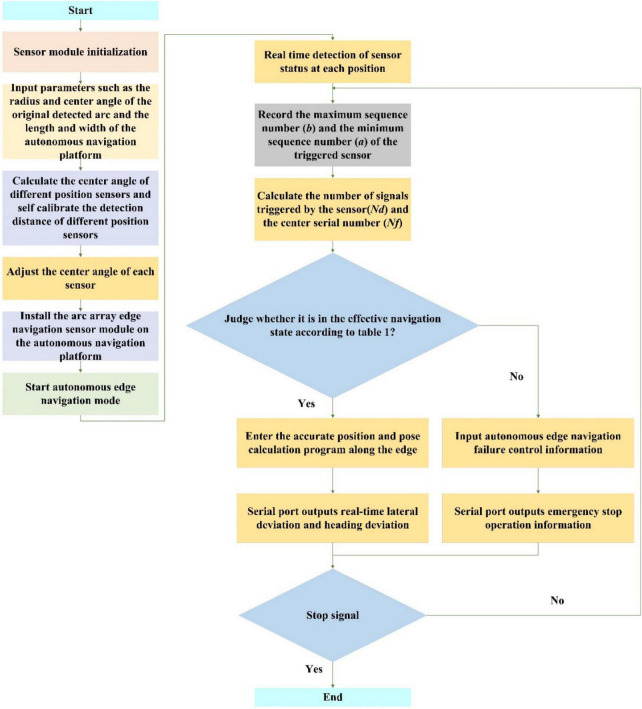
A software program flow of arc array edge navigation sensor module.

In this paper, the arc array edge navigation sensor module completes the detection and output of the relative position and attitude of the autonomous navigation platform through integrated packaging. It is necessary to carry out a complete process packaging design for the software program of the sensor system, as shown in Figure 20. The sensor system software program follows the sequence of key parameter setting, manual adjustment of the center angle of sensors in different positions, synchronous ranging of multiple ultrasonic sensors, calculation of relative roadside position and attitude state and output of position and attitude state signal execution. Finally, the modular design of the autonomous edge navigation sensor is realized. The edge-navigation method based on the principle of the circular array can be controlled according to the lateral deviation and heading deviation of the autonomous navigation platform relative to the road edge, and, also, according to the nine position and attitude states of the autonomous navigation platform relative to the road edge in Table 1. In order to ensure the safety of navigation along the border, the conditions not within the scope of Table 1 are considered as invalid conditions, which are used as the judgment of alarm signal output. In order to facilitate the user to detect the control signal output from the edge navigation sensor module of the arc array, the establishment data communication format shown in Table 3 is established. The data format uniformly adopts the hexadecimal compressed *BCD* code. The specific design is as follows: sxxxyy, sx is the symbol bit (sx = 00 indicates that the lateral deviation and heading deviation of the autonomous navigation platform relative to the edge of the road are positive, sx = 10 indicates that the lateral deviation and heading deviation of the autonomous navigation platform relative to the edge of the road are negative), xx represents integer digits, yy represents decimal digits, and the data content is the horizontal deviation and heading deviation of the autonomous navigation platform relative to the wayside. Where the 0th byte is 0 × 01 represents the frame identifier of the pose parameter message, the 1st, 2nd, and 3rd bytes represent the lateral deviation of the autonomous navigation platform relative to the curb, the 4th, 5th, and 6th bytes represent the heading deviation of the autonomous navigation platform relative to the curb, the 7th byte represents the status of the autonomous navigation platform relative to the curb, and the 8th byte is the checksum, which is used to verify the integrity and accuracy of the data.

**TABLE 3:** A relative wayside pose information data format.

Name	Identifier	Lateral deviation of relative road edge			Course deviation of relative road edge			Pose type	Checksum
Bytes	0	1	2	3	4	5	6	7	8
Definition	0 × 01	sx	xx	yy	sx	xx	yy	xx	CRC

## Experiment

**FIGURE 21:**
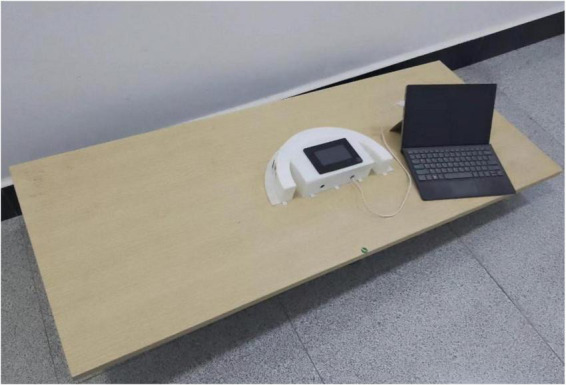
Performance testing.

In order to verify the rationality and effectiveness of the arc array edge navigation sensor module, a detection arc (Ju et al., 2017) was built on the autonomous navigation platform with a length of 1,200 mm and a width of 600 mm to carry out different feature detection arc impact tests, environmental factor impact and comparison tests, bump obstacle impact and comparison tests, and dynamic detection performance and comparison tests so as to detect the error of heading deviation. The horizontal deviation detection error and variation coefficient are analyzed as evaluation indicators, as shown in Figure 21.

### An arc influence test with different features

According to Equations (1) and (2) in this article, the position and orientation detection accuracy of autonomous curb line navigation is related to the setting parameters of the number of ultrasonic distance sensors (*N*_*C*_), the detection arc radius (*R*), and the central angle (θ). In order to clarify the influence of key layout parameters on the accuracy of the position and orientation detection, furtherly, the number of photoelectric switches is selected as 5, 7, and 9, the detection arc radius is 3,300, 4,300, and 5,300 mm, and the central angle is 5°, 10°, and 15° as the test levels; under the setting conditions that the detection arc radius is 3,300 mm and the central angle is 5°, the number of photoelectric switches is 7 and the central angle is 5°, the number of photoelectric switches is 7 and the detection arc radius is 3,300 mm. The forward normal condition (lateral deviation *D* = 0 mm, heading deviation γ = 0°) in Table 1 is used to test the detection accuracy of the edge pose affected by a single factor in the stationary state of the autonomous navigation platform.

In order to meet different accuracy requirements of autonomous navigation along edges, and to facilitate users to choose a circular arc array layout that meets the actual requirements. The number of ultrasonic ranging sensors, the radius of circle arc and the central angle of circle are taken as test factors. Similarly, under normal working conditions, the number of ultrasonic ranging sensors is 5, 7 and 9. The radius of the detected arc is 330 mm, 4300 mm and 5300 mm, and the central angle is 5 degrees, 10 degrees and 15 degrees as the test level. The multi-factor coupling influences the accuracy of position detection along the edge.

### A test on abrupt change of a concave convex along the road surface

Aiming at the problem that the photoelectric ranging sensor is easy to suffer from poor accuracy caused by environmental factors, such as light, reflecting surface color, and wall material, and to ensure that the detection distance of sensors at different positions can be independently calibrated to construct the detection arc requirements, the system selects the ultrasonic ranging sensor to develop the circular arc array edge navigation sensor. Because the ultrasonic distance sensor is a non-contact and wear-free detection of the detected object by using the acoustic medium, it has the characteristics of high frequency, short wavelength, and a small diffraction phenomenon. It can detect transparent or colored objects, metallic or non-metallic objects, solid, liquid, powdery substances, especially objects through which light cannot pass; the detection performance is hardly affected by any environmental conditions. Therefore, environmental factors, such as illumination, reflector color, and wall material are not considered to affect the performance of the circular arc array edge navigation sensor. However, there will be bump changes in the actual road edge plane. At this time, the effective pose reflected by the arc array edge navigation sensor deviates from the actual situation. In order to verify the influence of bump mutation along the road plane on the detection effect of the autonomous navigation platform on the edge position and attitude state, a detection arc with radius *R* = 3,291 mm and the center angle θ = 20° and chord length *L* = 1,200 mm (Ju et al., 2017) was constructed with seven ultrasonic ranging sensors. The position and attitude detection performance of concave and convex changes along the road was tested respectively according to the positive normal condition in Table 1 as the initial position and attitude state.

### Dynamic detection performance test

In order to verify the dynamic pose detection performance of the small integrated arc array edge navigation sensor module designed in this study, a detection arc with radius *R* = 3,291 mm and the center angle θ = 20° and chord length *L* = 1,200 mm (Ju et al., 2017) was constructed with seven ultrasonic ranging sensors. The test is carried out according to the independent position and attitude detection along the edge by walking in a straight line at the speed of 0.15, 0.25, and 0.35 m/s under the positive position and normal condition in Table 1. According to the triangular geometric relationship between the detection arc and the autonomous navigation platform, it can be known that the distance from the center point of the autonomous navigation platform to the edge under the positive position and normal condition is 300 mm. In order to ensure the repeated measurement accuracy and eliminate the random error in the measurement process, repeat the test for five times, take the average value of the test results, and analyze the stability.

## Results

### A test of the influence of the arc on different features

**FIGURE 22:**
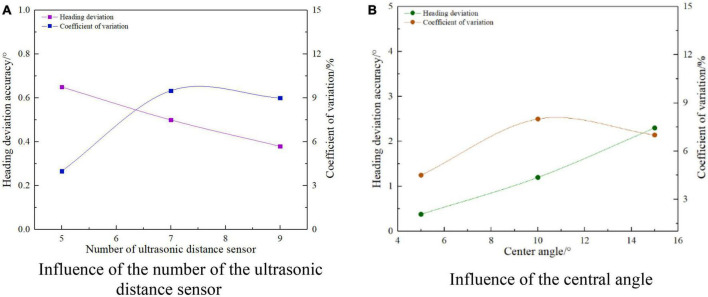
A single factor affects the accuracy of heading deviation detection.

It can be seen from Equations (1) and (2) that only the number of ultrasonic distance measuring sensors and the influence of the center angle are analyzed for the course deviation detection performance relative to the road edge. Figure 22 shows the influence of ultrasonic ranging sensor number and the center angle on the detection performance of heading deviation with a relative road edge. It can be seen from Figure 22A that the detection error of relative wayside heading deviation is not more than 1°, and the coefficient of variation is less than 10%. When the center angle is fixed, the detection error of relative wayside heading deviation decreases with the increase of the number of ultrasonic-ranging sensors, and the minimum detection error of relative wayside heading deviation is 0.4°. It can be seen from Figure 22B that the detection error of the relative wayside heading deviation is less than 3°, and the coefficient of variation is not greater than 8%. When the number of ultrasonic ranging sensors is fixed, the detection error of the relative wayside heading deviation increases with the increase of the center angle. When the center angle is 15°, the maximum detection error is 2.3°. To sum up, the number of ultrasonic ranging sensors is negatively correlated with the detection error of relative wayside heading deviation, and the center angle is positively correlated with the detection error of relative wayside heading deviation, which is consistent with the change of position and attitude detection deviation accuracy reflected in formulas (1) and (2). Therefore, under the condition of meeting the travel control time response, the number of ultrasonic ranging sensors should be increased and a small center angle should be set.

**FIGURE 23:**
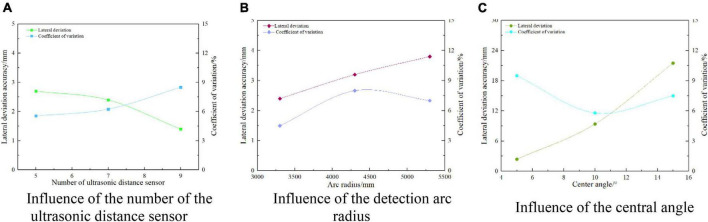
A single factor affects the accuracy of lateral deviation detection.

As shown in Figure 23, it is the result of the influence of the number of ultrasonic distance measuring sensors, arc radius, center angle, and the transverse deviation detection performance of the relative edge. It can be seen from Figure 23A that the transverse deviation detection error of the relative road edge is less than 3 mm, and the coefficient of variation is not greater than 9%. When the arc radius and the center angle are fixed, the transverse deviation detection error of the relative road edge decreases with the increase of the number of ultrasonic distance-measuring sensors, and the minimum transverse deviation detection error is 1.4 mm. It can be seen from Figure 23B that the detection error of the transverse deviation of the relative path is not more than 4 mm, and the coefficient of variation is less than 8%. When the number of ultrasonic distance-measuring sensors and the center angle is fixed, the detection error of the transverse deviation of the relative path edge increases, with the increase of the arc radius. When the arc radius is 5,300 mm, the maximum detection error has reached 3.8 mm. It can be seen from Figure 23C that the transverse deviation detection error of the relative road edge is not more than 30 mm, and the coefficient of variation is less than 10%. When the number of ultrasonic distance-measuring sensors and the arc radius is fixed, the transverse deviation detection error of the relative road edge increases, with the increase of the center angle. When the center angle is 15°, the maximum detection error is 21.5 mm. To sum up, on the premise of meeting the response accuracy requirements of autonomous navigation walking control, the ultrasonic ranging sensor should be appropriately increased, and the smaller arc radius and the center angle should be selected.

The performance of relative wayside heading deviation detection is related to the number of ultrasonic distance sensors and the center angle, while the performance of relative wayside lateral deviation detection is related to the number of ultrasonic distance sensors, arc radius, and center angle. Therefore, firstly, the influence of the number of ultrasonic distance-measuring sensors, the center angle, and the relative wayside heading deviation detection performance is analyzed, and then the influence of the number of ultrasonic distance-measuring sensors, the arc radius, the center angle, and the relative wayside lateral deviation detection performance is analyzed. As shown in Table 4, in order to further analyze the effect of the number of ultrasonic ranging sensors and the center angle coupling on the detection performance of the relative wayside heading deviation, SPSS software is used to fit the performance equation of relative wayside deviation detection affected by two factors. As shown in Equation (6), *R*^2^ after linear fitting of relative wayside deviation detection error and wayside deviation variation coefficient is 0.905 and 0.708, respectively. The fitting of relative wayside deviation variation coefficient is not significant, but the regression of relative wayside deviation detection error fitting model is significant, which can better describe the test results.

**TABLE 4:** Multi-factor coupling affects the accuracy of heading deviation detection.

Serial number	Influence factor		Course deviationγ/°	Coefficient of variation/%
	Total number of ultrasonic distance sensor/pcs	Central angle /°		
1	5	5	0.63	4.0
2		10	1.25	5.5
3		15	1.90	4.5
4	7	5	0.40	9.5
5		10	0.80	8.0
6		15	1.25	9.0
7	9	5	0.32	9.0
8		10	0.63	9.5
9		15	0.94	9.5

Further analyze the effect of the number of ultrasonic distance-measuring sensors, arc radius, and center angle coupling on the detection performance of the lateral deviation of the relative road edge, as shown in Table 5. SPSS software is used to fit the performance equation of three factors affecting the detection of the lateral deviation of the relative road edge, as shown in Equation (7). After linear fitting, *R*^2^ of the detection error of the lateral deviation of the relative road edge and the variation coefficient of the course deviation are 0.935 and 0.373, respectively. The regression of the fitting model of the measurement error of the lateral deviation of the relative road edge is significant, indicating that it is consistent with the actual situation and can better describe the test results, but the fitting of the variation coefficient of the lateral deviation of the relative road edge is not significant, The reason for this result is that the lateral deviation detected by the arc array edge navigation method is a fluctuation range, but it will not affect the actual navigation effect. To sum up, we can predict the autonomous edge navigation detection accuracy of different feature detection arcs according to Equations (6) and (7). Users can meet the needs of different autonomous edge navigation accuracy by arranging different feature detection arcs.


(6) $$\begin{array}{c} \Delta \gamma =-158\times 10^{-3}N_{t}+91\times 10^{-3}\theta +1.091\\ {\text{R}}^{2}=0.905\\ \Delta \gamma_{CV}=1167\times 10^{-3}N_{t}+17\times 10^{-3}\theta -0.722\\ {\text{R}}^{2}=0.708 \end{array}$$

**TABLE 5:** Multi-factor coupling affects the accuracy of lateral deviation detection.

Serial number	Influence factor			Lateral deviation γ/mm	Coefficient of variation/%
	Total number of ultrasonic distance sensor/pcs	Central angle/°	Arc radius/mm		
1	5	5	3300	2.77	5.50
2			4300	3.61	4.35
3			5300	4.45	5.00
4		10	3300	10.86	6.50
5			4300	14.15	4.85
6			5300	17.44	7.00
7		15	3300	24.32	6.00
8			4300	31.70	4.85
9			5300	39.07	5.00
10	7	5	3300	2.41	7.40
11			4300	3.14	8.00
12			5300	3.87	7.00
13		10	3300	9.47	7.50
14			4300	12.34	6.95
15			5300	15.21	7.25
16		15	3300	21.22	8.35
17			4300	27.65	9.00
18			5300	39.07	8.50
19	9	5	3300	1.41	6.35
20			4300	1.84	6.00
21			5300	2.26	5.95
22		10	3300	5.53	6.50
23			4300	7.20	7.00
24			5300	8.88	7.50
25		15	3300	12.40	6.25
26			4300	16.13	6.00
27			5300	34.10	6.00

In the formula: Δγ— relative along the road heading deviation detection error, °; *N*_*t*_—The number of ultrasonic ranging sensors; θ—Central Angle, °; Δ*γ_*CV*_*—The variation coefficient of relative roadside heading deviation detection error, %.


(7) $$\begin{array}{c} \Delta D=-1628\times 10^{-3}N_{t}+2443\times 10^{-3}\theta +4\times 10^{-2}R-16.907\\ {\text{R}}^{2}=0.935\\ \Delta D_{CV}=236\times 10^{-3}N_{t}+49\times 10^{-3}\theta -6.339\times 10^{-5}R-4.02\\ {\text{R}}^{2}=0.373 \end{array}$$

In the formula: Δ*D*—Lateral deviation detection error, mm; *R*—Radius of an arc, mm; Δ*D*_*CV*_— Lateral deviation detection error variation coefficient, %.

### A test on abrupt change of a concave convex along the road surface

Tables 6, 7 show the testing performance test of a normal road edge plane turning to a concave or a convex road edge plane. It can be seen from Table 6 that, when the normal road edge plane turns to the concave road edge plane, the transverse deviation detection error relative to the road edge increases by about 10 mm and stabilizes at about 45 mm, and the heading deviation detection error relative to the road edge increases by 1° and stabilizes at about 5°. It can be seen from Table 7 that, when the normal road edge plane turns to the convex road edge plane, the transverse deviation detection error of the relative road edge increases by about 8 mm, which is stable at about 40 mm, and the heading deviation detection error of the relative road edge increases by 0.5°, which is stable at about 4.5°. It can be seen from Tables 6, 7 that, when the normal path edge plane turns to the concave or convex path edge plane, the relative path edge attitude can maintain high-precision stable output for more than 30 s.

**TABLE 6:** Test results of detection performance of a normal road edge plane turning to a concave road edge plane.

Serial number	Normal road edge plane				Normal road edge plane turning to concave road edge plane					
	Accuracy of lateral deviation		Accuracy of heading deviation		Accuracy of lateral deviation		Accuracy of heading deviation		Time of duration	
	Error mean/mm	Coefficient of variation /%	Error mean/°	Coefficient of variation /%	Error mean/mm	Coefficient of variation /%	Error mean/°	Coefficient of variation /%	Time of mean/s	Coefficient of variation /%
1	30	6.50	3.5	9.46	40	9.30	4.5	8.50	40	7.90
2	28		2.8		45		4.0		45	
3	28		2.8		40		5.0		40	
4	30		2.5		50		4.0		35	
5	25		3.0		40		4.5		40	
Mean	28.20	/	3.03	/	43	/	4.4	/	40	/

**TABLE 7:** Test results of detection performance of a normal road edge plane turning to a convex road edge plane.

Serial number	Normal road edge plane				Normal road edge plane turning to convex road edge plane					
	Accuracy of lateral deviation		Accuracy of heading deviation		Accuracy of lateral deviation		Accuracy of heading deviation		Time of duration	
	Error mean/mm	Coefficient of variation /%	Error mean/°	Coefficient of variation /%	Error mean/mm	Coefficient of variation /%	Error mean/°	Coefficient of variation /%	Time of mean/s	Coefficient of variation /%
1	30	7.63	3.5	9.08	45	4.41	4.0	8.53	45	8.45
2	28		3.5		40		5.0		38	
3	28		3		42		4.3		35	
4	30		2.8		45		4.5		38	
5	25		3		43		4.0		40	
Mean	31	/	3.16	/	43	/	4.36	/	39.2	/

Tables 8, 9 show the testing performance test of a concave or convex road edge plane turning to a normal road edge plane. It can be seen from Table 8 that, when the concave changed road turns to the normal road edge plane, the transverse deviation detection error of the relative road edge recovers to about 35 mm, and the heading deviation detection error of the relative road edge recovers to about 3.5°. It can be seen from Table 9 that, when the convex changed road turns to the normal road edge plane, the transverse deviation detection error of the relative road edge recovers to about 30 mm, and the heading deviation detection error of the relative road edge recovers to about 3°. From Tables 8, 9, it can be seen that, when the concave or convex road changes from the plane to the normal road edge plane, the relative road edge attitude can be restored to the detection state of the normal road edge plane within 5 s. To sum up, the arc array edge navigation sensor module can stably output high-precision position and attitude data for more than 30 s after turning from the normal road edge plane to the concave or convex road edge plane, which ensures the safety of autonomous edge navigation, effectively reduces the probability of collision accidents, and has certain fault tolerance and good anti-interference performance. The arc array edge navigation sensor module can quickly restore the normal position and attitude detection state after changing from the concave or convex path edge plane to the normal path edge plane, which has good real-time performance.

**TABLE 8:** Test results of detection performance of a concave road edge plane turning to a normal road edge plane.

Serial number	Concave road edge plane turning to normal road edge plane					
	Accuracy of lateral deviation		Accuracy of heading deviation		Time of recovery	
	Error mean/mm	Coefficient of variation/%	Error mean/°	Coefficient of variation/%	Time of mean/s	Coefficient of variation/%
1	35	8.48	3.0	8.13	3.0	8.13
2	28		2.8		2.5	
3	28		2.5		3.0	
4	30		3.0		2.5	
5	30		2.5		2.8	
Mean	30.20	/	2.76	/	2.76	/

**TABLE 9:** Test results of detection performance of a convex road edge plane turning to a normal road edge plane.

Serial number	Convex road edge plane turning to normal road edge plane					
	Accuracy of lateral deviation		Accuracy of heading deviation		Time of recovery	
	Error mean/mm	Coefficient of variation/%	Error mean/°	Coefficient of variation/%	Time of mean/s	Coefficient of variation/%
1	35	9.08	3.5	8.48	2.5	6.50
2	30		3.0		3.0	
3	35		2.8		2.8	
4	28		3.0		3.0	
5	30		2.8		2.8	
Mean	31.6	/	3.02	/	2.82	/

### A dynamic detection performance test

**FIGURE 24:**
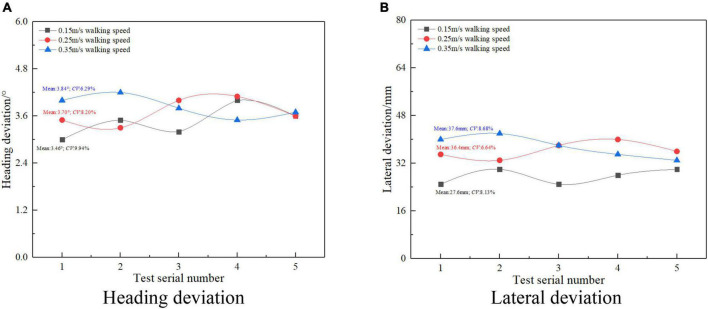
Dynamic test results.

Figure 24 shows the dynamic detection performance results of the small integrated arc array edge navigation sensor. The detection error of relative course deviation along the road is less than 4.5°, the coefficient of variation is less than 10%, the maximum coefficient of variation is 9.94%, and the minimum coefficient of variation is 5.59%. The detection error of lateral deviation of a relative road edge is less than 40 mm, the coefficient of variation is not more than 9%, the maximum coefficient of variation is 8.67%, and the minimum coefficient of variation is 6.64%. When the moving speed of the autonomous navigation platform increases from 0.15 to 0.35 m/s, the average detection error of the relative wayside heading deviation increases by 0.38°, and the average detection error of the relative wayside lateral deviation increases by 10 mm. As the walking speed of the autonomous navigation platform increases, the detection error of the relative wayside lateral deviation heading deviation increases slightly, but the variation coefficient of the relative wayside lateral deviation heading deviation is less than 10%. The performance of pose state detection is stable and real-time, which is not easily affected by the walking speed change of the autonomous navigation platform.

In addition, compared with Jia et al. (2015), to realize the edge detection method of a flat greenhouse road based on two-dimensional laser radar, the small integrated arc array edge navigation sensor is only affected by the unevenness and position mutation of the road edge plane, and has good adaptability to the gentle slope and uneven road surface on both sides of the road. Compared with the method of using machine vision to realize autonomous navigation path extraction (Wang et al., 2012; Hiremath et al., 2014; Malavazi et al., 2018; Yang et al., 2018; Inoue et al., 2019; Xu et al., 2021), the small integrated arc array edge navigation sensor uses ultrasonic distance sensor to detect the edge on the basis of the relative edge pose detection method based on the principle of the ideal target zone of the lateral center arc array. It avoids the influence of environmental factors, such as light, color of a reflecting surface, and wall material, and has good anti-interference ability. At present, the edge navigation method of a single ultrasonic or infrared photoelectric sensor (Feng et al., 2012; Zhao et al., 2012; Zhou, 2014; Wang and Liang, 2015) and linear arrangement of multiple ranging sensors to form an array module (Du, 2010; Xu et al., 2010; Yuan and Li, 2013) cannot realize the accurate position and attitude feedback of the autonomous navigation platform, and it is difficult to ensure the smoothness and control accuracy of the edge navigation. However, a small integrated arc array edge navigation sensor based on the principle of ideal target zone of lateral central arc array – the construction of the relative edge pose detection method can realize accurate pose state detection, and can improve the smoothness and stability of an autonomous navigation platform.

## Conclusion

Based on the ideal target band principle of the lateral central arc array, this paper studies the relative edge pose detection method, and constructs a universal relative edge pose detection model with unknown number of sensors. Aiming at the sore point that the current arc array is too large to be integrated, a small integrated arc array navigation sensor module is designed by reducing the layout radius of the ranging sensor, adjusting the center angle of the non-equal circle and increasing the detection distance. The main conclusions are as follows:

(1) This research has developed a small integrated arc array navigation sensor module with a cost of about US $100, which can accommodate at least nine ultrasonic ranging sensor groups, and proposed an autonomous construction method for detecting arcs with different characteristic parameters based on adaptive calibration of detection distance so as to improve the convenience and friendliness of users, and, at the same time, it can meet different requirements for autonomous edge navigation. It can also meet the requirements of low-cost, high-precision, and fast border navigation in greenhouse, animal, and plant factories and other environments.
(2) The experimental results of arc detection with different features show that the accuracy of the edge position and orientation navigation method based on the arc array is related to the key layout parameters of the detected arc. With the increase of the number of ultrasonic distance-measuring sensors, the detection errors of the heading deviation and the lateral deviation of the relative road edge are reduced. The detection accuracy of the heading deviation of the relative road edge is increased to 0.4°, and the detection accuracy of the lateral deviation of the relative road edge is increased to 3 mm. With the reduction of the center angle, both the heading deviation and the lateral deviation detection error of the relative curb decrease. When the center angle is 5°, the heading deviation detection accuracy of the relative curb reaches 0.38° and the lateral deviation detection accuracy of the relative curb reaches 2.4 mm. With the reduction of the arc radius, when the arc radius is 3,300 mm, the lateral deviation detection accuracy of the relative curb reaches 2.4 mm, and the heading deviation detection accuracy of the relative curb is not affected. Therefore, when setting the detection arc, the number of ultrasonic distance-measuring sensors should be appropriately increased, and the smaller arc radius and the center angle should be selected. In addition, the number of ultrasonic distance sensors, arc radius, and center angle significantly affects the detection accuracy of lateral deviation and heading deviation relative to the curb. Through the linear fitting equation, the prediction regression equation of the number of ultrasonic distance-measuring sensors and the heading deviation detection accuracy of the center angle and the relative edge is obtained. The prediction regression equation of the number of ultrasonic distance-measuring sensors, the circular arc radius, and the horizontal deviation detection accuracy of the center angle and the relative edge is obtained. The *R*^2^ factor of the linear fitting is 0.905 and 0.935, respectively, which has a high fitting reality; this equation can be used to predict the detection accuracy of the detection arc along the edge with different layout feature parameters so as to quickly select the layout scheme suitable for the actual needs.
(3) The experiment on abrupt change of a bump on the road edge plane: when facing the operation environment of concave change and convex change on the road edge plane, when turning from the normal road edge plane to the concave change road edge plane or convex change edge plane, the transverse deviation detection error relative to the road edge increases by 10 mm, which is stable at about 45 mm; the heading deviation detection error relative to the road edge increases by 1°, which is stable at about 5°; and the relative road edge attitude can maintain high-precision and stable output for 30 s. The arc array edge navigation sensor module has certain fault tolerance.
(4) The dynamic detection performance test results show that, when the arc radius is 3,291 mm and the center angle of the circle is 20°, and the traveling speed of the autonomous navigation platform is 0.15 to 0.35 m/s, the detection errors of the lateral deviation and the heading deviation relative to the road edge are less than 40 mm and 4.5°, respectively. As the traveling speed of the autonomous navigation platform increases, the average detection error of the relative road edge heading deviation increases by 0.38°, and the average detection error of the relative road edge lateral deviation increases by 10 mm. However, the coefficient of variation is less than 10%. The dynamic position and attitude detection performance of the arc array edge navigation sensor module is relatively stable and has good real-time performance. It is less affected by the walking speed change of the autonomous navigation platform, and can be used for autonomous edge navigation control.

The small-scale integrated photoelectric arc array edge navigation sensor studied in this paper uses the ultrasonic distance sensor to establish the detection arc. Because the transmitted signal of the ultrasonic distance sensor is a divergent conical detection surface, the reflected signals of the adjacent ultrasonic distance sensors will interfere with each other, resulting in large errors in the detection process. The plane position of the ultrasonic distance sensor can be reasonably arranged to solve the problem of mutual interference between adjacent ultrasonic distance sensors. In addition, the ultrasonic ranging sensor will be affected by temperature, humidity, air pressure, air flow, and other factors, which will reduce the ranging accuracy and cause large errors in the position and attitude of the relative roadside. Subsequently, the influence of the above environmental factors can be weakened through the temperature and humidity compensation algorithm to further improve the adaptability of multi agricultural scenarios.

## Data availability statement

The original contributions presented in this study are included in the article/supplementary material, further inquiries can be directed to the first author (BX; 2668804078@qq.com).

## Author contributions

BX, JL, and LC designed the study, conducted the trials, and analyzed the data. BX wrote the manuscript. YL provided test prototypes. HJ and LL polished the English grammar of the manuscript. All authors contributed to the article and approved the submitted version.
